# Ghost Hunting in the Broken Archives: Re-Historicizing Digital Education in an Institutional Context

**DOI:** 10.1007/s42438-022-00330-3

**Published:** 2022-08-16

**Authors:** Michael Gallagher, Stuart Nicol, Markus Breines

**Affiliations:** 1 Centre for Research in Digital Education, University of Edinburgh, 34 Canaan Lane, Edinburgh, EH10 4SU UK; 2 Educational Design and Engagement, Learning, Teaching and Web Services, University of Edinburgh, Edinburgh, UK; 3 London School of Hygiene and Tropical Medicine, Keppel Street, London, WC1E 7HT UK

**Keywords:** Digital education, Autoethnography, Hauntology, Broken archives

## Abstract

Digital education is often presented as breaking from tradition. A failure to account for how digital education emerges from historical institutional activity is problematic insofar as this activity continues to circulate through the present and future, appearing and disappearing in often unexpected ways. Using Derrida’s hauntology as a theoretical lens, this paper traces how a digital education initiative at the University of Edinburgh in 2003 carried through to the creation of a course to train teachers to teach online in 2019, which in turn informed the university’s response to the pandemic in 2020. Working in a broadly autoethnographic way alongside archival document analysis, several findings emerged. First, hauntology provides a mechanism for institutions to trace their own histories and to note how these histories, often hidden in archives or carried forward into the present by hosts, inform their present and future trajectories. Second, broken archives, those that have ceased to function as active repositories but are disconnected from institutional domains and ontologies, shut due to absent gatekeepers, or merely forgotten, contribute​ to the sudden and often unexpected emergence of hauntings in present and future trajectories. Third, curation of the archive is an act of reinterpretation, one that troubles historical narratives and introduces new hauntings. All these findings assert a re-historicizing of digital education by emphasising the hauntings from the past that inform its emergent present and contested future, countering many of the ahistorical imaginaries of digital education.

## Introduction

Digital education is often framed as a break from historical university practice and an alignment towards more market-driven discourses around personalisation, efficiencies, and scale, and towards the artefacts of those discourses such as microcredentials and teaching excellence frameworks. Yet, the history of digital education is not a recent development nor an ahistorical anomaly, rather an ongoing focus as ‘histories … stretch back at least as far as the birth of computer science and cybernetics in the 1940s’ (Williamson and Eynon 2020: 223). Ultimately, as stated by Papert (1987: 23), these discourses ‘betray a tendency to reduce what are really the most important components of educational situations—people and cultures—to a secondary, facilitating role. The context for human development is always a culture, never an isolated technology.’

More and more of the work of teaching is distributed through the broader teacher function (Bayne 2015) that ensemble of teacher-student agency, broader networks of professional staff, computation, and code. Additional elements that inform this teacher function include policy, strategy, compliance, and student satisfaction, much of which intentionally or otherwise conspires to ‘unbundle’ (Czerniewicz et al. 2021) not only the teacher function, but also the historical institutional memory that supersedes it. In this ‘break’ from tradition towards a more distributed form of digital education, the narratives of how institutions arrived *here* fail to shape how they might go *there*.

In response, this paper explores how elements of past digital education at the University of Edinburgh, bound in uncertain or broken archives, re-emerged during a period of institutional instability and contributed to an emergent space marked by ‘abrupt and discontinuous movements’ (Law and Mol 2001: 615). We surface how particular actors and past institutional activity in digital education acted as hosts and haunted the present. This was marked both by the historical experience of the institution which was ‘simultaneously kept hidden from view and made visible’ (Good 2019: 411) in various forms, and its mutability as artefacts from the past were reintroduced, surfaced, and submerged. This paper situates this discussion historically in the institution in the period beginning in 2003, the beginning of the Principal’s eLearning Fund (PeLF) project and ending in 2020, and the initial digital responses to the Covid-19 pandemic.

Within that period, the institution has invested in several successive strategic projects, amongst other related activities, to build research-focussed capacity in digital education. The Principal’s eLearning Fund (PeLF) project ran between 2003 and 2008 and was intended to be very broad and ‘ground-up’ in scope, touching on many aspects of teaching practice and what was referred to as e-learning (Anderson et al. 2009). One area that grew quickly was online, or distance, education, which delivered on one of the original drivers for PeLF: that the institution remains competitive in an increasingly globalised, and privatised, education marketplace. Following from PeLF, investment was made specifically in online programmes, with a distance education initiative which ran from 2010 until 2014. Further investment was initiated in 2012 to grow a portfolio of Massive Open Online Courses (MOOCs) (Haywood et al. 2013). Partnerships were made with external providers Coursera, FutureLearn, and edX in 2012, 2013, and 2016. Further investment was made in 2018 to investigate distance learning at scale, or the feasibility of delivering masters degrees, and microcredentialed micromasters degrees, at scale with external partners (University of Edinburgh 2019). The staff development course discussed here was part of this final investment, immediately predating the enforced investment in online teaching caused by emergency responses to the pandemic.

Strategically, there has been a consistent investment in digital education since 2003; however, differing models of support for these initiatives, compounded by the inevitable changes over 19 years in the senior staff responsible for them and the platforms and mechanisms used to support them, have led to breakages and gaps in approaches and practices. Some initiatives have been held in institutional consciousness more than others, whilst technical archiving of artefacts has been inconsistent and under-addressed. PeLF, perhaps by virtue of being the oldest, but perhaps also because of its wide-ranging nature, seems least remembered and therefore more ghostly as we have unearthed its artefacts in autoethnographic work. It is for this reason we focus primarily on artefacts from that era, linking to those most recently developed during the pandemic.

This paper notes how artefacts emerging from PeLF to build capacity in digital education in 2003 can be traced through to the creation of an online course designed to train university staff to teach online in 2019. This course, Edinburgh Model for Teaching Online, created pre-pandemic, became a core component of the overall university response to the pandemic pivot online. The paper draws heavily on archival information and autoethnographic data from the authors to demonstrate how past institutional activity around digital education informed the institutional present. We are positioning the legacy of institutional activity around digital education to be the substance of this haunting, which was drawn on to inform the emergent spaces of the larger institution during a period of crisis.

Hauntology represents a broad range of approaches all drawing focus on the return or persistence of artefacts from some cultural past. Hauntology refers to a ‘haunting’, or an encounter with something that is of the past that re-emerges in the present, often abruptly. Discussed by Derrida in *Spectres of Marx* (1994), hauntology allowed for a critique of anti-Marxist attitudes and modern-day capitalism emerging in the wake of the collapse of the Eastern Europe Communist Bloc. Taken in its original form, hauntology was a critique of the failure to ‘exorcize the spirit of Marxism’ (Bullen et al. 2006: 55) from modern and late-stage capitalist discourse. Derrida returned to the subject of haunting in *Archive Fever* (1996) to note how these older hauntings are bound in archives that are added to with new artefacts and hauntings as those who engage with them produce ‘more archive, and that is why the archive is never closed. It opens out of the future.’ (Derrida 1996: 45)

Hauntings are characterised by three traits. The first being that a haunting emerges and re-emerges often in a chronologically unexpected fashion as it is ‘not dated, it is never docilely given a date in the chain of presents, day after day, according to the instituted order of a calendar’ (Derrida 1994: 3). Hauntings can be sudden depending on the audience that are receiving them. Those familiar with their origins will receive them as predictable progressions; indeed, Derrida (1996: 9) noted the origin of the word archives (*arkhé*) as meaning both *commencement* and *commandment.* For those unfamiliar with their *commencement* nor with the authority with which they speak (*commandment*), these hauntings appear suddenly, often without provocation.

Second, hauntings are products of our (institutional, individual, societal, historical) selectivity; we must choose what artefacts to pull forward into the present and those that we do not. ‘We must never hide from the fact that the principle of selectivity which will have to guide and hierarchize among the “spirits” will fatally exclude in its turn … this watch itself will engender new ghosts.’ (Derrida 1994: 109) This second trait foregrounds the importance of archives and that ongoing process of selectivity and how we (institutions, sectors, societies) are creating further hauntings in these selections.

Third, hauntings are necessary elements for defining future trajectories as ‘without this non-contemporaneity with itself of the living present … without this responsibility and this respect for justice concerning those who are not there, of those who are no longer or who are not yet present and living, what sense would there be to ask the question “where?” “where tomorrow”’ (Derrida 1994: xviii). As such, these hauntings are necessary for understanding our current and future trajectories.

Hauntings can surface how certain actors and artefacts appear and disappear as ‘unseen (others) maintain the seen’ (Postma 2020: 132). These ‘others’ refer just as equally to the ‘missing masses’ (Latour 1992) of non-humans as to humans; technological infrastructures, governance, policy and strategy language, data, and more create contingencies and ‘flicker’ often unexpectedly between states of absence and presence. It is the haunting of various historical actors, human and non-human alike, that allows for a critical examination of how institutional responses to the present and future are haunted with institutional artefacts, bound in broken archives, and mobilised in spaces marked by ‘mutable chaos’ (Diken 2011). This theoretical approach allows us to trace these hauntings from their origin to its emergence in the more recent present.

The methodological framework presented includes autoethnography and textual analysis. We pair our autoethnographic accounts and their tracing of hauntings with textual analysis of archival documents, particularly the final evaluation report of the project under investigation that serve to further cohere this emergent space and the artefacts and actors haunting it. Artefacts in this instance refer to the outputs and archival documentation of PeLF, and actors refer to the people involved in PeLF and an Edinburgh Model for Teaching Online course, many of which still work in the university to this day. The overall framework is one that gives ‘ontological primacy, not to groups or places, but to configurations of relations…the point of fieldwork becomes to describe a system of relations’ (Becker 1996: 56) that defines this emergent space and through which these hauntings circulate.

Three themes emerged from this analysis. First, we consider the trace data that surrounds the project; second, we note the necessity of hosts, human or archival, in carrying forward the hauntings into the present; and third we consider how these hosts and hauntings converged on an Edinburgh Model for Teaching Online course, which was taught to 600 staff at the university during the spring and summer of 2020. By exploring the trace data that surrounds the project, the hosts carrying forward the hauntings into the present and how these converged on an Edinburgh Model for Teaching Online course, this paper considers human-digital assemblage of permissions and gatekeeping emerging historically and continuing to shape the present. This process of re-historicizing digital education provides new insights and problematizes many of the ahistorical imaginaries of digital education.

## Situating this in the Postdigital Archive: Ghost Hunting, Interpretation, and Hauntology

The archive is a violent initiative of authority, of power; it's taking power for the future, it pre-occupies the future; it confiscates the past, the present, and the future. We know very well that there are no innocent archives. (Derrida 2001: 85)

In this paper, we draw on literature related to archives that ‘prosthetic’ of ‘individual and cultural memories and identities’ (Anderson 2021: 106). We explore the role that broken archives play in shaping institutional trajectories through present and future contexts. Broken archives are those that have ceased to function as active repositories but are disconnected from institutional domains and ontologies, shut due to absent gatekeepers, or merely forgotten. They contribute​ to the sudden and often unexpected emergence of hauntings in present and future trajectories. Archives are fluid, highly selective, and ultimately political bodies as its ‘archiving structure is one that is essentially ‘privileging’ and hence ‘patriarchal’ (Anderson 2021: 107). Its selective structure, what Derrida (1996) refers to as the finitude of the archive and its ‘violent selectivity’ (Naas 2014: 28), is a precondition of its existence. Anderson (2021: 108) extends this precondition further:

An archive is only recognisable because it is finite; and it is finite because for ‘reasons both contingent and necessary, one cannot keep or save everything’. The archive is structured by the choices around what we save and keep and what we don’t (and ‘repression’ as Derrida shows us in Archive Fever, would be no simple form of forgetting or destroying—if that were indeed possible—but an archiving form of or process of ourselves). (Anderson 2021: 108)

It is defined as much by what is excluded as what is selected; as such, the histories contained in these archives are never comprehensive in their composition; they are ‘partial and fragmented accounts’ (Deepwell 2020: 253). They are interpretable, and there is power in those that can interpret them, despite the accessibility of this interpretability as ‘no discipline or sector of culture has a monopoly on potential analyses, much less a monopoly on answers’ (Derrida 1994: viii). Blackman (2019a: 41) argues that this selectivity is compounded in the digital, where ‘both science and computational culture are haunted by both the histories and excesses of their own storytelling’ and that these excesses surface in ‘haunted data to be mined, poached and put to work in newly emergent contexts and settings’.

Yet, the digital archive is precarious in that it is finite but voluminous and contingent on a host of digital and cultural structures. The removal of these structures creates what we refer to as broken archives. Broken archives are those that have ceased to function as active repositories but are disconnected from institutional domains and ontologies, shut due to absent gatekeepers or curators, or merely forgotten or rendered opaque due to a missing contingency. Digital projects end, curators are reassigned, staff retire, and the digital traces of that activity are abstracted in folders, sites, and platforms. The brokenness of these archives is ultimately a condition of its existence as ‘digital environments repurpose and refashion the logic of the archive under conditions of social, political, ecological, and technological uncertainty’ (Agostinho et al. 2019: 423). This uncertainty and the overall archival ‘vulnerability’ (423) contribute​ to the often-unexpected emergence of hauntings in the present and future trajectories as ‘the refuse of that broken archive’ is mined into ‘another set of perspectives and possibilities’ (Chambers 2019: 21). As such, we see the archive as Derrida (1996) does: as ‘affirmation of the future to come’.

We position the digital archives as a site of ‘ghost-hunting’ (Blackman 2019b) to be ‘mined’ for later use for ‘opportunities’ in emergent contexts. Its interpretability is bound in itself and in other hosts, which in this case are the people with an understanding of how the archive, broken or not, came to be. They are often gateways for how the archive is to be interpreted, and they host the hauntings themselves. Hauntology represents a broad range of approaches all drawing focus on the return or persistence of artefacts from a cultural past. It is premised on an ‘indeterminate relationship between “then” and “now”, “present” and “absent”, “being” and “non-being”’ (Bozalek et al. 2021: 1). It advances, among many other things, the logic of the ghost who continues to haunt the present and future from the past. Fisher suggests that a haunting ‘refers to that which is no longer, but which is still effective as a virtuality’ (Fisher 2012: 19). Hauntology engages with the future as well, as an ‘idea of the future from the past haunts the present as if it were here now’ (Stock 2021: 148). As such, projections of the future from the distant past resurface in the present and continue to inform thinking, even as merely a ‘virtuality’.

Hauntology has been used broadly across a range of fields, including providing a mechanism for balancing critical textual readings and more speculative approaches in literacy and linguistic studies (Davis 2005); for providing a strategy for creating spaces for past histories in oppressive societal contexts in arts and media studies (Harris 2015); as a means of understanding cultural memory through monuments (hardware), texts (software), and specters (ghostware) in history (Etkind 2009); as a practice for surfacing ‘the complex processes through which traumatic dimensions of contested historical experience are simultaneously kept hidden and made visible’ in psychological anthropology (Good 2019); and as a means of interrogating the sociopolitical nature of data-driven infrastructures (Dixon-Roman 2017). All of these studies illustrate how hauntings from within these fields reverberate into the present and future. Educationally, hauntology has been used to critique higher education policy reform (Brøgger 2014), and as a pedagogical framing particularly for forgotten or troublesome concepts and histories (Zembylas 2013). Geerts (2021: 1) suggests that the pandemic crisis demands a ‘pedagogical but also ethico-political reorientation toward the hauntological powers of past-present-future injustices…’. In this case, hauntology becomes a mechanism for acknowledging that the ‘the principle of selectivity’ which originally guided and hierarchized the creation of the archive itself, also ‘fatally’ excluded other ghosts (Derrida 1994: 109). Hauntology becomes, potentially, a reparative act of acknowledging those who were excluded from the ‘violent selectivity’ (Naas 2014: 28) of the archive in its commencement.

Moreover, hauntology offers resistance to the rhetoric and commercial imaginaries associated with the digital, engaging critically with their ‘totalising and universalistic claim and the tendency towards finality and inevitability that seems to cancel historicity’ (Brøgger 2014: 523). Hauntology provides a means of evading being locked into ‘atemporal and ahistorical analyses’ (Dale and Robertson 2012: 27). Again, in the digital, we see the projections of the future appearing from the past, as ‘what haunts the digital cul-de-sacs of the twenty-first century is not so much the past as all the lost futures that the twentieth century taught us to anticipate’ (Fisher 2012: 16). Hauntology offers a means of tracing how artefacts circulate through the often volatile spaces of the present by turning our critical gaze to their historical origins, by avoiding tendencies towards discursive accounts of digital inevitability, and by reclaiming past projections of the future.

## The Institutional Context: Principal’s eLearning Fund (PeLF) and Edinburgh Model for Teaching Online

The institution under investigation is the University of Edinburgh. It currently has around 40,000 students and 15,000 staff, organised into three colleges—Arts, Humanities, and Social Sciences; Medicine and Veterinary Medicine; and Science and Engineering. Schools comprising aligned discipline areas sit within each of these colleges, made up largely of academic departments and administrative centres. The university has more than 70 fully online programmes with over 4000 students alongside a large MOOC infrastructure that has had over four million enrolments since 2013. This body of work in digital education is underscored by the university’s interest in using the growing portfolio of online learning programmes to innovate new approaches to learning and teaching. Indeed, in policy and strategy, ‘being digital is portrayed by the institution as inherently positive, requiring transformation from an inferior, pre-digital state’ (Fawns 2019: 135). The context discussed here was shaped in many ways by the broader interest at the University of Edinburgh, codified in policy and strategy, that largely attempts to move away from this ‘pre-digital state’.

In this paper, we are using PeLF and one training course created in 2019 as the objects of analysis, but we are broadly referring to the legacy of infrastructure, research and development projects, collective teaching expertise and practice, policy, and governance at the university supporting digital education since the mid-2000s. This activity is not solely ‘static and singular, the sum of recorded files and learned procedures’ but rather ‘dynamic stories’ residing in people and ‘dispersed across the array of actors that make up the differentiated polity’ (Corbett et al. 2018: 1). These stories and the knowledge bound within them is fluid, moving between actors and elements in mutable ways, rarely fixed aside from its more material manifestations such as technological infrastructure and policy.

Much of the capacity for this digital education can be traced to the Principal’s eLearning Fund (PeLF), an initiative which began in 2003 and concluded in 2008. PeLF was a major, time-limited initiative designed to foster the development and widen the use of e-learning in the University of Edinburgh. During this time, funding of circa £3.6 million pounds was allocated to the fund (2003–2004, £400 K; 2004–2005, £800 K; 2005–2006, £1.2 million; 2006–2007, £800 K; 2007–2008, £400 K), which sponsored 641 projects in total through a process of annual, competitive bidding. The types of projects funded through this scheme varied in scope. They range from modest enhancements to existing courses to make them more digital to the creation of new, entirely online, programmes. Of the 641 projects funded through this scheme, many were focused on similar aspects of education: digitally enhanced assessment and feedback, e-portfolios, and fully online programmes featured prominently. All the schools of the university, except for one, received funding from PeLF. Distribution of projects across the three colleges was more even than might be expected in a competitive bidding scheme. There were a number of cross-school projects within individual colleges, but little work was done across colleges in an interdisciplinary fashion. It is through PeLF that we seek to explore the ways that specific hauntings from 2003 to 2008 were carried through to the present and recirculated through the university in 2019 and 2020 (Table 1).

**Table 1:**
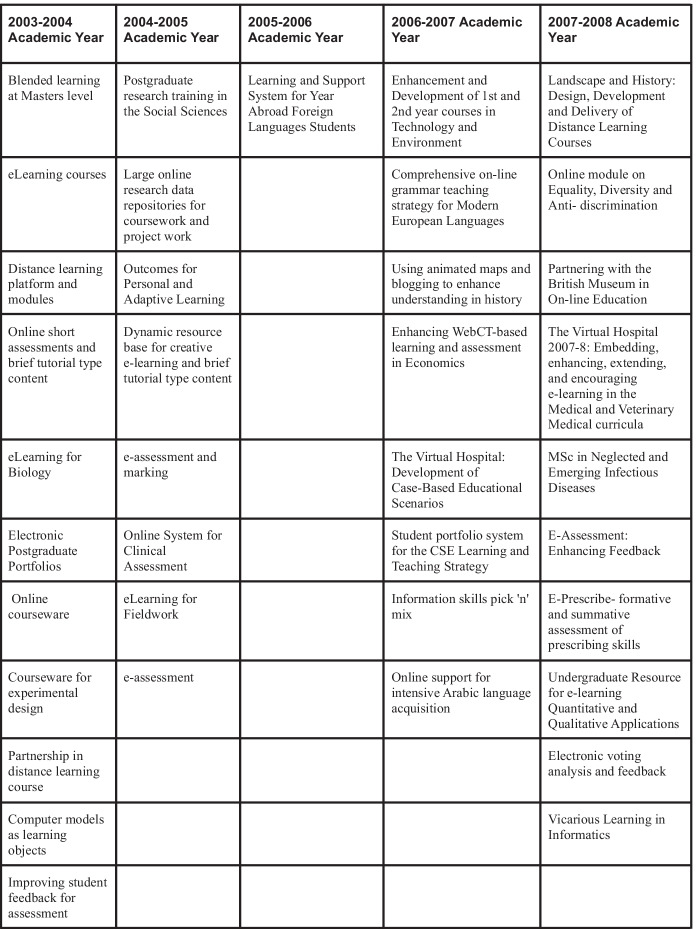
Initiatives funded by the PeLF project, many of which carried in some form into the present (Anderson et al. 2009)

An Edinburgh Model for Teaching Online is a 7-week course for those new to teaching online at the University of Edinburgh. It was created in 2019 by a team including the authors responding to a scenario, whereby many online tutors would need training in these new forms of online teaching (Gallagher 2019). Since March 2020, it has run three times to a total of 700 staff, first in response to the Covid-19 pandemic from April to August 2020, and then again in August 2021. It is an experiential online course designed to allow staff to actively engage in online education as a student might, to reflect on that process, and adapt teaching practice as a result. It advanced a specific pedagogy in the process, one that emphasised engaged online teachers, engaged learning communities, and authentic and actionable feedback and assessment. The course exposed staff to a variety of spaces that digital education might inhabit such as blogs, discussion boards, multimedia platforms, synchronous applications, and collaborative online spaces.

Originally designed for the edX platform in 2019, the course was quickly moved to the university supported Learning Management System (LMS) Blackboard Learn in March 2020 ahead of the beginning of the April 2020 run of the course. Without such a move, the course would be rendered institutionally insignificant as the university emphasised the use of university-supported technologies during this period. edX was outside the scope of that support. Thus, the rapid adaptation and movement of the course from edX to Blackboard Learn in March 2020 placed it within the university ‘infrastructure which permits it to happen’ (Duffy et al. 2020: 3). This course is significant only insofar as it hosted many of the hauntings emerging from PeLF and became part of the university’s pandemic response to preparing staff to teach online for the 2020–2021 academic year. The themes presented in the following section speak to how past university activity—the digital hauntings of the institution—emerged in this course host and was amplified in crisis.

## Methodology: Defining the Spatial and Spectral Terrain

In this paper, we are taking a broadly autoethnographic approach along with archival analysis as the primary methods for this study. Autoethnography captures the embeddedness of the authors themselves in this haunting: We are haunted by institutional activity around digital education, and we co-designed and taught the aforementioned course. It relies on personal experiences to ‘foreground how meaning is made among people occupying and connected to digital spaces’ (Dunn and Myers 2020: 43). The authors were and continue to be actors in this institutional context; our personal experiences, coupled with archival information, form the substance of the recollections presented in this paper and are attempts to ‘create evocative and specific representations of the culture/cultural experience’ (Adams et al. 2017: 3). Yet autoethnography requires working at the intersection of both itself and ethnography, to ‘create a representation of cultural practices that makes these (institutional) practices familiar for cultural “outsiders” (3). To do this, we set out in this study to position ourselves as participant observers. We observed the hauntings that this paper describes, traced them historically in archival documents, and, in our own accounts, noted their circulation through the larger university in the present. Further, we reflected on our own responsibility in acting as ‘hosts’ of the hauntings, carrying them into the present and prompting their return by embedding them in the Edinburgh Model for Teaching Online course.

Autoethnography is used across a range of interdisciplinary fields and presents value as a methodological approach to understand the ways in which digital technologies are changing how we live and work (Pink 2016). It is clear that ‘ethnography is not merely a useful tool to describe everyday practice; it is also playing a critical role in intervening in the world’ as it can ‘provide insight into motivations and practices that in turn shape future directions’ (Pink 2016: 6). We felt it is important to note the futuring dimensions of this context, that what was being mobilised in this case was not only a response to the chaotic present, but an acknowledgement of future intent. This ‘anticipatory knowledge’ (Nelson et al. 2008; Selin 2008) is a situated and spatial phenomenon that requires scrutiny as it intervenes in the hauntings discussed in this paper.

Further, defining the fieldsite as ‘a heterogeneous network’ (Burrell 2016: 56) presents an advantage for this study. Working in a broadly autoethnographic way, we are ‘able to show how individuals made sense of ambiguous … social terrain in the course of lived experience’ (Burrell 2016: 53). The hauntings discussed in this paper are examples of ambiguous space: orphaned web pages about the PeLF initiative; a final report left in an ambiguous state of closure on an institutional wiki; originality matches against the PeLF final report from Turnitin’s plagiarism detection database; and traces of project transactions in a finance system. Using hauntology alongside broad autoethnographic practice provides a means of defining the ‘spatial terrain where the social phenomenon under study took place’ (Burrell 2016: 51).

Our hauntings, examples from a long dead institutional initiative, provide ‘entry points’ to ‘multiple types of networks’ (Burrell 2016: 56) that enable the authors to ‘hunt’ the ghosts. Hauntology allows us to think beyond chronology and genealogy to conceive how the past exists in the present, and the future, within these heterogeneous networked field sites. The spatial terrain discussed in this paper is elicited in ‘embodied contexts of practice and everyday experience’ (Ito 1996); through co-occurring and ephemeral arrangements of modes (Dicks et al. 2006: 82); and by drawing on connective ethnographic practice where ‘activities and expectations about those activities’ are identified (Hine 2007: 620).

It helps to define, problematically in this case due to the interdependency of the myriad of actors involved, what is excluded from this study. We deliberately include a specific set of historical artefacts, a discussion of tracing them into the present, and the implications of this for institutional change. We exclude many more historical artefacts not due to the richness of possible analysis, but rather due to our position as participant observers within this context. Much of our discussion here is problematically based on the notion of ‘being there’ (Hannerz 2003: 202) ethnographically. The authors were very much embedded or emerged from the historical artefacts haunting the institution: Nicol joined the university as part of the PeLF haunting described in this paper, Gallagher is the Programme Director of an online programme that emerged from this same haunting, and all three authors co-created and taught the online course. Our reflexivity as participant observers is directed most comprehensively towards these specific hauntings. We note the need to be reflexive about ‘the ways in which we produce knowledge, the status of that knowledge, and subsequently what this tells us about what that knowledge can mean’ (163). Yet, while we do ascribe to Mol’s position (2002: 32) of ethnography as emphasising practice and its ‘everyday enactments’, we also note that it is indeed necessary to identify the historical origins of these practices and the hauntings embedded within them, and what broader impact they have had on the emergent spaces of the present in ‘diverse, scattered, contradictory sites and domains’ (Simon and Randalls 2016: 5). We also note the tension here in presenting this hauntology as we cannot ‘delicately render’ these accounts, surface ‘all of their complexities, nor situate in their particular contexts or day-to-day work’ (Simon and Randalls 2016: 6).

The data drawn on for this research spans two periods of time: the first, encapsulated in the final PeLF report (Anderson et al. 2009), details a digital education project at the university which ran from 2003 to 2008. The PeLF report is not currently available on a public website; however, several web pages are still referencing the initiative. This appears to be by accident rather than design. At the time of writing, these links are working; they may or may not be when you read this which further surfaces the way in which these hauntings flick between states of absence and presence.^1^

The second period of time involves our recollections detailing the events from 2019 to 2021 gathered in working in a broadly ethnographic way. Alongside ongoing correspondence between the authors from March 2020 to October 2021, much of this recollection was captured in October 2021 as the authors recorded their discussions about their arrivals at the university, and their attempts to trace the historical origin of the hauntings that informed the pandemic response at the institution. Further, gathering anecdotes provide a useful way of surfacing the role of non-human actors within research (Adams and Thompson 2016). These discussions were transcribed and thematically and inductively coded. The themes emerging from this activity were used to triangulate findings from archival research, most notably those from the PeLF final report. Notable passages from this data are used in this paper to frame discussions.

## Themes: Trace Data, Hosts, and Pedagogy

In keeping with Derrida’s hauntology and its evasion of being locked into ‘atemporal and ahistorical analyses’ (Dale and Robertson 2012: 27), we explore more fully the concept of trace data. Trace data is a digital data associated with institutional projects that continues to circulate well beyond the life cycle of the project. In the case of PeLF, this refers to, for example, long since used financial cost codes, broken links pointing seemingly to project workspaces, intranet and wiki pages unbound from any institutional directory or web ontology, adrift yet maintained by gatekeepers and hosts. We discuss the role of ‘hosts’, those artefacts, and actors that carry the hauntings and reinsert them into the institutional consciousness at seemingly sporadic intervals. We posit that without these hosts, the hauntings would cease to carry forward and, in effect, terminate. Artefacts refer to the outputs and archival documentation of PeLF, and actors refer to the people involved in PeLF and an Edinburgh Model for Teaching Online course, many of which still work in the university to this day. These actors and artefacts have some degree of overlap as many of the actors involved drafted or guided the PeLF project and the course.

## University of Edinburgh as a Broken Archive: Trace Data

With the particular digital form of hauntology being presented here in this paper, it is ‘necessary to speak of the ghost, indeed to the ghost and with it’ (Derrida 1994: xix). Much of this speaking to and with the archival ghosts that populate this paper is done through the surfacing of trace data that suggests its very existence in the first instance, and subsequently suggest the contours of its cascading impact on the present.

We turn to the trace data surrounding the PeLF final report (Anderson et al. 2009) and note the contingent assemblages of human and non-human actors that make access to archival information possible. Gathering anecdotes provides a useful way of surfacing the role of non-human actors within research (Adams and Thompson 2016). The following anecdote exemplifies how the authors learned of the PeLF final report as trace data, but also how a wiki, a technology platform, has been left to liaise with distant human hosts to make decisions about, and manage access to, material traces of that initiative.

I (Nicol) decide to spend time looking for half remembered traces of a course in half forgotten project archives. I find the report on a sprawling university-wide wiki, but only by using search terms drawn from my inside knowledge of the project. As I traverse the pages looking for more clues, I click a link about budgetary information. The wiki tells me I don't have permission. It's not really relevant so I think nothing more of it. Within an hour I receive an email from an ex-colleague who has long since moved to another part of the university. Without asking me the wiki has emailed them to ask if I can get access. They enquire why I want access and then decline the wiki's request made on my behalf (and without my knowledge). A couple of days later I receive an email from the wiki telling me that I have been granted permission by a different ex-colleague, no questions asked. Clearly the wiki had decided to ask more than one of the last known gatekeepers. (Nicol) 

We continue to find signs of the PeLF report in the trace data left behind by the project’s existence. We note the trace data lingering in external databases such as Turnitin, the plagiarism detection service. Turnitin frequently trawls the web taking copies of website content into its own databases at various points in time. The PeLF report matches against work submitted to Turnitin, demonstrating that the report was at one time publicly available. At some point, it ceased to be outwardly maintained, available only to those on the inside of the institution, and as the project wound down, essentially orphaned as it became linked to less and less of the university’s online navigation and sub-domains, and (hyper)linked to fewer and fewer subsequent digital initiatives at the university.

Yet there is trace data that remains which is not mirrored ‘reflections of human sociality’ but rather ‘ontologies that emerge from myriad intra-actions in a world that is always already in process of becoming’ (Dixon-Román 2017: 47). The trace data portends that this ‘becoming’ will keep ‘becoming’ well into the emergent present in which this paper is being written, heeding Pott’s (2021) call to the interpretability of the archive and how its ‘objects and documents are not just presented, but represented, interpreted, read or viewed in certain ways by both the curator and an audience’. We the authors are now curating the archive ourselves by piecing together this trace data and extracting significance from the final report. We are, in essence, haunting the institution alongside this PeLF project and its attendant trace data artefacts.

This curation is dependent on how the trace data, indeed all the archival materials, are inscribed in mediums that we can interpret in the present, fulfilling the duty of the archive to ‘offer itself, as the unique work that it is, to future iterations or repetitions or representations, interpretations, or readings’ (Naas 2014: 127) long after the creator, or in this case project team, is gone. Yet, as we discuss in the next theme, these creators are rarely fully gone; they continue to carry and curate these hauntings as hosts and circulate them in the emergent present.

## University of Edinburgh as Hosts for the Hauntings: People

...we've traced it back to the Principal's e-Learning Fund and my recollections of that being kind of more important about the people it drew in, rather than the projects that it spawned. (Nicol)

The hosts that have and continue to circulate these hauntings into our emergent present deserve further scrutiny. Hosts refer to human and non-human actors that carry with them the hauntings emerging from the PeLF project. For the human actors, in many instances, these were people brought into the university as a result of PeLF, or contingently drawn together around the project for its lifespan (2003–2008). Hosts, as vehicles for hauntings, provide ‘a way of thinking with and through dis/continuity – a dis/orienting experience of the dis/jointedness of time and space, entanglements of here and there, now and then, that is, a ghostly sense of dis/continuity’ (Barad 2010: 240). Although PeLF points to an institutional trajectory in relation to digital education, without hosts ‘there is no overarching sense of temporality, of continuity, in place’ (Barad 2010: 240). Hosts carry with them, curate, and circulate the hauntings that emerged from PeLF and carried through to the present. Despite the hauntings flickering between states of absence and presence in the present in sporadic, seemingly unpredictable ways, the hosts remain seemingly more steadfast in their temporality, continuity, and associations with artefacts of place. Fisher (2012), discussing the hauntological ‘experience of a time that is out of joint’ (20), points to ‘the way in which the past has a way of using us to repeat itself’ (19). People are characterised as key actors both in the processes of change and in its continuity and sustainability. We characterise this as the ability of people to act as hosts for hauntings, enabling persistence of past events to speak implicitly to the present and future. They are archives unto themselves, bearing with them understandings of both commencement and commandment.

The arrival of a new principal of the university in 2002 (the conceiver and champion of the PeLF programme of work), as a key disruptor of existing networks, drives the University of Edinburgh to become a ‘centre of excellence’ and ‘centre of innovation’ in ‘institution-wide implementation’ and ‘leading edge developments in learning and teaching through the use of e-learning’ (Anderson et al. 2009: 15). However, in the PeLF final report, we frequently noted ‘people’ in relation to the notions of ‘retention’ and ‘development’. The link between people and sustainability is exemplified by this passage:

It was recognised that PELF could have contributed to the ‘human capital’ of the University by allowing the acquisition of staff with specific areas of learning technology expertise. Accordingly, we gathered information on the backgrounds of, and the nature of the contributions made by, project staff with the aim of informing decisions on how best to deploy their expertise in the future and to build the career progression of this group of staff. (Anderson et al. 2009: 3)

The report notes that in 2009, ‘fifteen of the nineteen project staff interviewed are still employed here, in all but one case, very much with a strong e-learning focus’ (Anderson et al. 2009: 55). What is remarkable is that in 2022 we can identify at least 3 senior learning technology staff, including one author (Nicol), who were employed because of PeLF and have had the opportunity to grow a career in learning technology. Secondary influence can also be untangled through those who studied the MSc in E-learning (now the MSc in Digital Education), a PeLF-funded course, and have since taken up senior academic posts in the Centre for Research in Digital Education and throughout the university. This is the route taken by another author and co-designer of an Edinburgh Model for Teaching Online course (Nicol). In this sense, we the authors have acted as hosts, or actors of persistence in the university network, connecting PeLF with a pandemic response to teaching practice and carrying these hauntings forward, without being aware of our roles as hosts until writing this paper.

These hosts are defined as much by their capacity to actively carry forward these PeLF hauntings as by their restricting access to wider circulation, as was made evident in the trace data and archival discussion, specifically ‘how people still have access and are able to give access, but that's the only way to get to that archive as well, via those gatekeepers and those people’ (Nicol). The hosts act as gatekeepers and contribute to the survival of certain texts (De Vos 2020) by passing them along or limiting access to them in the first instance.

## Pedagogy as Hosts: What Constitutes a ‘model’ from the University of Edinburgh?

Hauntings from the PeLF past were embedded within an Edinburgh Model for Teaching Online course, which became part of the institutional pandemic response in 2020. Alongside the people involved acting as hosts, the course concepts themselves were hosts of the hauntings. This begins with the title of the course itself. The decision to call our staff development course ‘An Edinburgh Model for Teaching Online’ fell out of a conversation during the design sessions that preceded the course. It is a rather bold and somewhat contradictory statement suggesting both that there is something unique about the University of Edinburgh approach, but also that there is more than one. Although none of the authors was consciously aware at the time, there is a sense that PeLF, a long since discontinued initiative, reappeared or haunted our self-conscious naming of the course. The following statement shows the ambition of the previous Principal for University of Edinburgh to be unique:

At the first meeting on the 14th of January, 2003, the Principal noted how the existing models in UK universities for fostering e-learning could not readily be applied within University of Edinburgh and therefore that University of Edinburgh would need to find its own solutions. This initial declaration of the need to find ways ahead that would be well-tailored to the University of Edinburgh context, rather than import ready-made approaches from outside, may be seen as giving a powerful steer to subsequent decision-making in this area. (Anderson et al. 2009: 11)

There was also a sense of trepidation around the embedded multiplicity of disciplinary practice at the University of Edinburgh, coalescing around the three colleges. It appears that each college organically developed separate approaches to managing bids within the PeLF project. In fact, there is a pointed question from the external advisor to that PeLF project to consider if this ‘was one fund, or three’ (30). This approach to assume colleges act differently haunts our staff development course through the use of the indefinite article ‘An’. Fawns et al. (2021), critiquing subsequent initiatives at the University of Edinburgh, characterise this as structurally common to ‘ancient’ universities. However, the scale of online teaching practice encountered at University of Edinburgh is not common, and our self-conscious sensitivity to a multiplicity of ‘University of Edinburgh’ approaches to online teaching does appear unique and enduring.

This continued into the concepts underpinning the course, which was designed to frame online teaching practice around engaged teaching, community building, and responsive feedback and assessment. The course was designed to do so through a multitude of technologies supported by the university, providing a frame for participants in the course to experience online study as a student might, and to allow that experience to inform practice. Yet this emphasis on experientialism was itself a haunting emerging from past, largely PeLF activity and carried forward by hosts, including the authors Nicol and Gallagher as noted in their recollections:

Gallagher: This was a good part of what the course was about, and a lot of CPD (Continuing Professional Development) should be based on that kind of experience. But I don't remember what was the driving force...

Nicol: Well, I think it's that approach of the teacher getting the student experience. I suppose in my head, it had kind of been a mantra as part of the staff development approach that the new tutors should get the experience of being an online student and that's the best way for them to kind of learn and empathise with their own online students. But also you have the secondary thing that actually what they're learning about are all the approaches and technologies that they're going to use in their teaching.

Gallagher: I don't remember that coming up until later. But then when you track back a little bit and you saw that some of the stuff from past projects was emphasising experiential learning and we were like, Okay, that could be a happy accident, or it could be some of that was being filtered up through the memory somehow.

Nicol: Absolutely. The experiential one was, I think, one of several instances where we sort of discovered parts of the course that were emerging in other parts of the university and other time periods, from the past, somehow. It wasn't just the experiential part. Emphasis on a student centred kind of pedagogy and teacher presence.

An Edinburgh Model for Teaching Online course was repurposed for pandemic response in spring of 2020 and made available to all staff for the purposes of preparing for the next academic year. It was at this moment that the course was brought from its original ‘host’ edX into the university virtual learning environment (VLE), placing it within the university ‘infrastructure which permits it to happen’ (Duffy et al. 2020: 3). At this moment of repurposing and making it available, its role as a host to a multitude of institutional hauntings around digital education and online teaching was most pronounced. It became, in some ways, a spectral conduit from PeLF to the present.

We must note however that the university cannot be seen as a ‘static container’ (Bayne et al. 2014) with strict delineations of what is inside or out, but rather a fluid space of orchestration where ‘hosts, guests, buildings, objects, and machines are contingently brought together to produce certain performances in certain places at certain times’ (Hannam et al. 2006: 214). Moving the course to within the boundaries of the university was done contingently to enact a particular performance; without this movement within, the course itself might still be performative but would ultimately cease to be mobilising for the institution. The institutional hauntings that begat the course in the first instance would have been muted had it remained in this outside state. It would have ceased to exist as a spectral apparition, particularly during the volatility of the institutional pandemic response.

Within this re-emergence of the course and the 600 staff that engaged with it during the spring and summer of 2020, hauntings hosted therein re-emerged and were amplified broadly and at times problematically across the institution particularly during the pandemic response. We acknowledge that this course is now an archive itself, one that both curates a broken archive and harbours a myriad of ghosts that will, however modestly, haunt some institutional future. The participants who took the course will act, or not, as hosts themselves exerting some measure of control over present and future institutional trajectory. As Derrida (2010: 68), the authors see this as an ‘affirmation of the future to come’ and an act of ‘saying yes to all those others and interpretations that we don’t know and can’t control’.

## Identifying the Ghosts and its Implications for Digital Education and Institutional Practice

These hauntings reveal several points of note for institutions. First, they are firmly situated historically and hosted in the artefacts and actors of the institution. Second, they never fully exit the institutional consciousness but are remade and recirculated at intervals. Subsequently, they demonstrate that a full departure from our institutional past is both unlikely and unnecessary. It is ultimately a trajectory on which to ground institutional futures. The implications of these hauntings and this research for institutional practice fall into several categories.

The first is that hauntology provides institutions utility in understanding how past activity informs present and future trajectory. Hauntology rehistoricizes the present and future by re-engaging with the ghosts that circulate through it, ghosts that can be traced to archives and people. Any engagement with the present and future is, when seen through the lens of hauntology, an historical one. The archive itself is critical here as ‘the archive therefore is the haunting of past and future, which means that culture can never be fixed and absolute, but is always dynamic, responsive, and affecting. The future always already haunts the past and vice versa’ (Anderson 2021: 111). This archive is not merely dormant, waiting patiently for hosts to re-engage with it, but rather acts in the present, which in turn is ‘troubled by the agency of the past’ (Brøgger 2014: 522). Engaging with archives becomes an institutional practice designed to understand and exert some measure of control over the present and future trajectory.

This archival work is what Derrida (1996: 68) referred to as ‘affirmation of the future to come’. Derrida positions a lack of control in the archive to its interpretability; each future ‘curation’ of the archive interprets the present and future from it. These interpretations will naturally vary due to a host of contingencies that have assembled around that curation. Our presentation of the PeLF hauntings and an Edinburgh Model for Teaching Online course are contingent to our position as researchers, as hosts of hauntings in and around digital education at the institution, and in 2022 at the time of writing as increasingly distanced from the urgency of the early pandemic response. These interpretations would likely change with different curators, in different times, and with different objectives. That variability is to be welcomed as the underlying culture is never ‘fixed and absolute’ but always ‘dynamic, responsive, and affecting’ (Anderson 2021: 111). Using hauntology as a lens to engage with the archives as a means of understanding present and future trajectory is, or can be, a rich institutional practice.

Hauntology as a means of interrogating past institutional activity allows for analysis as to what ‘ghosts’ are being created in the present that will continue to haunt the future. An Edinburgh Model for Teaching Online course, contingent as it was to the institutional pandemic response, congealed around it a collection of hosts from throughout the university who designed and taught on the course. This was noted in the autoethnographic data in this passage from Nicol:

I think the course was just very, very well-positioned, and partly, or mainly, because of the people…We were there in the place to be able to put the resources in place to make all the underpinning stuff happen. So we could quickly get the course migrated across, get some people to help with the teaching on it, and all those kinds of bits. Essentially how a good relationship between an academic development unit and an information services team should work. (Nicol)

The relationships between these units, and amongst the larger pool of teachers drawn from across the university to teach on the course, created a new contingency through which hauntings circulated. Institutionally, there are implications here for practice, particularly in and around the drawing together of teams for large institutional projects or more urgent responses to present circumstances. Noting the proclivity of these teams to act as hosts, to curate archives, and to cascade hauntings into the future, the selection of members is critical.

This leads to a further implication for institutional practice, namely, how universities are often loosely coupled organisations with often weak internal links. Loosely coupled systems refer to a combination of autonomy (loose) and interdependence (coupling) between different elements of the institution, e.g., educational programmes, faculty development units, senior leadership, and project teams (Fawns et al. 2021). As such, the contingencies that allow for hauntings to occur and hosts to circulate them are often precariously constructed. This precariousness, reflected in the following passage from the report, emphasises the fragile contingencies of hosts, control, localised innovation, and archival responsibility (positioned as project management, coordination, duplication, resources).

The distributed, very loosely coupled, approach placed ‘considerable responsibility for project management in the hands of local innovators’ (op.cit., p.12). It involved ‘a range of units offering support on the basis of their expertise and location in the university’ (ibid.). This approach was regarded as offering ‘an economical solution to the provision of support resources without imposing unnecessary controls on innovative teaching projects (op.cit., p.13) but as raising issues concerning the coordination and management of project activities, the potential for innovations to falter and the duplication of effort and resources’. (Anderson et al. 2009: 10)

In adapting hauntology to the digital and noting its utility in understanding how digital artefacts from the past are carried into the present and future, we present another implication for institutional practice. In rehistoricizing the present and future, we also inevitably trouble the narratives of the past, accounts of how we as an institution got *here*, a point which Carstens (2021: 120) draws alongside the uncanny: ‘As a thoroughly uncanny epistemological engagement that illuminates the present's problems as it approaches the future, hauntology requires us to trouble official narratives of how we got ourselves into this mess.’

We troubled the accounts bound in the PeLF final report noting how and when they faltered or failed to circulate as hauntings at a later time or failed to be hosted in people at the institution. We observed how some found their way to an Edinburgh Model for Teaching Online course and in turn the institutional pandemic response. We noted how the broken archives contributed to the suddenness of how these hauntings appeared and disappeared. This troubling, we argue, is both necessary and a rich institutional practice, a process by which we re-interpret and curate the past through the lens of the present. In such practice, the past is never static, but rather a dynamic and malleable resource.

There are limitations of this approach, largely due its institutional scope and scant claim to comprehensiveness. This is an account from a single institution and is very selectively presented in terms of tracing the hauntings emerging from one project in 2003 to one course in 2020. It does so with a limited dataset, relying on the final report from the PeLF project alongside accounts from actors who had little agency in the events their accounts are detailing. The broken archives that we drew from to inform this approach was broken at least partly because of our doing; our ‘violent selectivity’ (Naas 2014: 28) in what we chose to include makes its subsequent interpretation more ‘partial and fragmented’ (Deepwell 2020: 253). Yet, we would argue that this is a precondition of archival work itself and hunting the origins of the ghosts that emerge from them. We trouble the past to find our ghosts in the present and future, if only partly to note that this same past is writing our present and future.

The broader significance of such an approach is the utility it provides in conducting layers of analysis across a considerable timespan. In the case of this research, it is across two decades. Such approaches can be used more broadly for understanding how digital education ripples into emergent spaces, ones characterised by crisis. Hauntology provides a lens for macro levels of analysis to explore sector level discourses and the carrying forward of ghosts contained therein. Mesa level institutional analyses of the sort provided here provide utility for understanding how past activity and investments ripples into the institutional present and future. Hauntology provides a means for reclaiming and rehistoricizing the future, to fully engage with the position that ‘universities need to get better at crafting their own, compelling counter-narratives concerning the future of technology in teaching, to reassert the agency and presence of the academic and student body in the face of technological change’ (Bayne and Gallagher 2021: 608). These counter-narratives, we argue, are informed by the ghosts around us, the hosts that harbour them, and the archives that cry out for curation and interpretation.

## Footnotes

1 The site of the Principal’s eLearning Fund project, active as of July 2022: http://www.elearn.malts.ed.ac.uk/pelf/index.html. Accessed 18 July 2022.
